# Efficacy of Faculty Development Training Workshops (FDTWs) on Writing High-Quality Multiple-Choice Questions at Northern Border University (NBU) in the Kingdom of Saudi Arabia (KSA)

**DOI:** 10.7759/cureus.62607

**Published:** 2024-06-18

**Authors:** Anshoo Agarwal, Osama Khattak, Safya Ebraheem Esmaeel, Eslam Fahmy, Naglaa Ahmed Bayomy, Syed Imran Mehmood, Hamza Mohamed, Abdulhakim Bawadekji, Fahad Abdullah J Alotibi, Malek Saad M Alanazi, Abeer Younes

**Affiliations:** 1 Pathology and Laboratory Medicine, Faculty of Medicine, Northern Border University, Arar, SAU; 2 Restorative Dentistry, Jouf University, Sakakah, SAU; 3 Medicine, Northern Border University, Arar, SAU; 4 Physiology, Northern Border University, Arar, SAU; 5 Anatomy, Northern Border University, Arar, SAU; 6 Biological Sciences, Northern College for Nursing, Arar, SAU; 7 Medicine, Faculty of Medicine, Northern Border University, Arar, SAU

**Keywords:** medical faculty, assessment, feedback, efficacy, item analysis, mcq, quality, multiple-choice questions, training workshop, faculty development

## Abstract

Background: A multiple-choice question (MCQ) is a frequently used assessment tool in medical education for both certification and competitive examinations. Well-constructed MCQs impact the utility of the assessment and, thus, the fate of the examinee.

Aims and objectives: To analyze the basic science faculty perceptions of writing high-quality MCQs, to create awareness of item-writing flaws in constructing high-quality MCQs, and to determine the impact of faculty development training workshops (FDTWs) on MCQ writing skills.

Material and methods: An online workshop was held over two weeks for basic science faculty to learn high-quality MCQ construction. Faculty-made MCQs were analyzed for flaws, and a questionnaire assessed the impact of the workshop on MCQ construction. Pre- and post-workshop responses were compared to evaluate the necessity of such workshops for improving faculty skills in MCQ assessments.

Results: A total of 47 (83.2%) of participating faculty believed the workshop could reduce MCQ construction errors. The participants agreed that a series of workshops were needed for lasting improvements in MCQ construction.

Conclusions: One-day short-duration workshops, such as the current one alone, cannot achieve the objectives of training participants to write high-quality MCQs. To improve student assessment through high-quality MCQs, the faculty needs to be exposed to continuous and frequent sessions that will help them.

## Introduction

Multiple-choice questions (MCQs) are among the most accepted forms of student assessment in medical education. MCQ assessments present several advantages, such as the ability to help evaluate the learning levels of a vast number of students in an expansive topic range under shorter durations [1]. In general, MCQs are mostly employed for summative assessments of students and trainees in medical colleges [2]. However, they can be used for both formative and summative analyses [1]. A recent study concluded that most medical students prefer MCQ-based assessments over other methods [3].

Preparing a competent MCQ paper is a time-consuming and intensive task. Preparing a competent MCQ paper is time-consuming because it requires aligning questions with learning objectives, designing complex questions to test different cognitive levels, avoiding common flaws, balancing difficulty and discrimination, and ensuring validity and reliability. Key steps include defining objectives, writing and reviewing questions, pilot testing, and iterative improvement. The MCQ papers written by teachers and educators should be reliable and in line with the objectives of the given curriculum. Thus, medical educators need to be adroit in designing constructive test materials that challenge students’ thoughts and learning processes [4].

The aim of any type of student assessment should not be merely factual recall but to understand higher-order cognitive skills. Higher-order cognitive abilities should be assessed using application-based questions, analysis, synthesis, and evaluation. Case studies and real-life problem-solving situations promote critical thinking and knowledge application beyond factual recall [5]. High-quality MCQs can improve student performance [6], deviate from the accepted guidelines, and alter the discrimination index (DI) and difficulty index (P-value), thereby impacting student scores and the validity of the exam itself [7]. The most commonly occurring item writing flaws (IWFs) when designing an MCQ tend to be the usage of terms such as ‘except’, ‘not’ etc., and a vague or unrelated STEM [8].

Constructing a well-framed MCQ is one of the important tasks undertaken by medical teaching faculty, and it is necessary for them to be aware of the established set of indexes and guidelines to be followed while writing MCQs. In reality, we find that most writers are often unaware of them or lack the suitable training needed to put these guidelines into practice. A high-quality MCQ-based assessment while remaining unbiased can help understand student progress, learning processes, and thinking capacity while allowing the educator to discern the capacity of each student [9,10].

Item analysis is one of the most important characteristics of MCQ writing. Without proper item analysis, the question itself can offer clues to the correct answer, thus negating its validity in testing students’ ability to solve problems and think critically. This means that the results of the assessment are unreliable and cannot be used to differentiate between high and underachievers [4].

Medical faculty, while being experts in their respective fields of specialization, might not have proper training in academic skill development. This is why faculty development training workshops (FDTW) are essential for teaching faculties. The FDTW on the writing of MCQs can benefit both faculty and students. Such FDTWs have been shown to result in meaningful improvements in participants’ knowledge. In addition, quality-determining factors, such as group interactions and discussions, dedicated practice sessions with feedback, and study material, play an important role in the successful outcome of any such workshops [11]. FDTW constituting exercises, presentations, printed guidelines, and training sessions can help improve the ability of instructors to design technically accurate MCQs void of common flaws that render them invalid [12]. A cross-sectional study that analysed 1,202 MCQs designed to assess fourth-year clerkship medical students reiterated the importance of training and encouraging faculty members to design and build MCQs to attain higher cognitive levels [13].

Without proper training, educators create questions that are poor in quality and lack the capacity to properly test students’ cognitive levels [14]. Questions that carry bias or flaws can lead to either bumped-up or misconstrued results [15]. High-quality MCQ assessments can help students opt for a deep learning approach, which is highly beneficial [16]. Irrespective of the academic or professional background of the participants, attending FDTWs contributed to their experience in a constructive manner [17].

Studies detailing the effect of FDTWs on MCQ writing among medical educators have been conducted, and the outcomes have been extensively published. FDTW can be single, short durational, or longitudinal. Both have been found to be effective in their own ways. Owolabi et al. [18] established the need for continuous training and retraining of educators to improve MCQ quality, focusing on the impact of longitudinal FDTW on MCQ item writing [18]. Gupta et al. [9] reported that a short durational course might be insufficient to bring about the required improvement the in quality of MCQs and recommended long durational training for maximum effectiveness. However, Sezari et al. [14] suggested that one-day workshops could result in a fundamental improvement in the capacity of attendees.

Salih et al. [19] found that the quality of MCQs improved considerably after the medical education department adopted the guidelines for MCQ writing. They observed a marked reduction in item flaws, better difficulty factors, DI, and functionality of distractors [19]. Shaikh et al. observed improvement in the item analysis skills of participants after attending FDTW [20].

Several factors can influence the outcomes of FDTW. These include interest in the participants, previous training sessions attended, exposure to reading material, level of seriousness, and renewed interest in the concept being elaborated. A study conducted on the impact of workshops on topics pertaining to medical education technologies concluded that knowledge levels increased immediately after attendance, decreasing at two months, and then reverting at six months [21]. This variation in the ability of participants to recall and utilize the information given to them through FDTW can be used to determine the frequency at which FDTW should be organized.

It is also essential to study the immediate effect of any FDTW conducted to understand the benefits of the reading material, group discussions, and brainstorming sessions. Feedback sought from the participants is generally generic, but a questionnaire designed to assess the individual needs of the attending member could actually help improve future sessions [22].

Aim and objective

The aim of the study was to analyze the perception of writing high-quality MCQs among the basic science faculty of the Medical College of Northern Border University, to create awareness about item-writing flaws in constructing high-quality MCQs among the basic science faculty of the medical college of Northern Border University. The study also aimed to determine the impact of FDTWs in improving high-quality MCQ writing skills among the basic science faculty of the medical college of Northern Border University.

## Materials and methods

Study design

A webinar and FDTW were conducted with the aim of exposing the participating faculty to designing MCQs for assessment, indicators of a high-quality MCQ, and the occurrence of IWFs while building high-quality MCQs. The workshops were organized and conducted by the Medical Education Department of Northern Border University, involving experts in medical education and faculty development. The research was an educational intervention run for six months, from September 2023 to February 2024, at Northern Border University in Arar, Saudi Arabia. Its goal was to assess how well basic science faculty members could create high-quality MCQs after attending an FDTW.

Participants' selection criteria

The inclusion criteria for the study comprise basic science faculty members of the Medical College at Northern Borders University, specifically those aged between 30 and 62 years, with educational qualifications including Bachelor of Medicine, Bachelor of Surgery (MBBS), Doctor of Medicine (MD), or Doctor of Philosophy (PhD) and possessing between five and 35 years of professional experience. The exclusion criteria exclude any faculty staff not part of the Medical College at Northern Borders University, as well as part-time and visiting faculty members. Faculty members who agreed to take part in the workshop and complete the pre- and post-workshop questionnaires were included in the research. Those who failed to finish either questionnaire were not excluded. Participants provided their informed permission, and Northern Border University's Bioethics Committee granted ethical clearance (research project number MEDA-2023-12-2356).

Data collection

The workshop comprised interactive sessions where faculty created and discussed MCQs to enhance their skills. Theoretical concepts and practical modules covered item analysis, MCQ structure modification, and adherence to guidelines. Participants who attended the MCQ Workshop as part of the FDTW were presented with two self-structured, pre-validated questionnaires: one to be answered before and the other after the FDTW (pre- and post-workshop questionnaires). These questionnaires were designed not for comparison between themselves but for pre- and post-workshop surveys of participants, based on earlier studies. They measured the research objectives both before and after the workshop session (pre-post format). The process involved identifying goals that required improvement, ensuring correlation between the goals and the questions, and applying an understanding of item writing defects to improve the alternatives list, lead-in, and stem in a logical order. The pre-workshop questionnaire, designed to gather insights into participants' knowledge of the general principles and guidelines of MCQ design and their attitudes towards the FDTW, was answered before the start of the workshop.

During the session, participants were divided into different groups, with each group being asked to assign a leader, scribe, and feedback presenter. Participants assigned leaders to each group based on specific criteria such as leadership experience, communication skills, or subject expertise. During the workshop brainstorming session, group discussions, presentations, and Q and A sessions were conducted. The attendees were first introduced to Bloom’s taxonomy and item anatomy. As the session progressed, they were asked to create levels C1 and C3 MCQ and analyze their work.

At the end of the workshop, participants answered a post-workshop questionnaire. The post-workshop questionnaire was designed to assess whether the MCQ workshop was useful and if it increased the attendees’ knowledge base. It also included questions to gauge the participants’ perceptions of the role of MCQs and how much the session had changed their outlook. Each question in both questionnaires was given five-point options such as ‘strongly agree', 'agree', 'neutral', 'disagree', and 'strongly disagree’, and the results were presented as percentages. All pre- and post-workshop MCQs thus generated were compiled, typed, proofread, and analyzed for the quality of the MCQs constructed.

Data analysis

Data were entered into Microsoft Office Excel and analyzed using Statistical Product and Service Solutions (SPSS, version 25; IBM Corp., Armonk, NY). Continuous variables were expressed as mean (standard deviation), and categorical variables were expressed as numbers and proportions. The chi-square test was used to compare categorical variables between the pre- and post-workshop MCQs. Nonparametric tests of significance were used to compare continuous variables as the data did not follow a normal distribution. P < 0.05 was considered as significant.

Theoretical background of this study

In medical education, MCQs are a popular and widely utilized way to examine the cognitive domain. There are numerous benefits to the MCQ format. It is possible to examine a large number of students at once and cover a larger topic range. MCQs can also evaluate students' higher-order cognitive abilities, such as knowledge application, analysis, and interpretation. Higher reliability and validity are found in MCQs of superior quality. They are adept at distinguishing between high and low achievers. The MCQs must be flawlessly constructed in order to accomplish this. The MCQs involve principles from learning theories, assessment theories, and psychometrics. MCQs should be reliable, valid, and fair, assessing various cognitive skills per Bloom's taxonomy. Effective design minimizes cognitive load, ensures clear unbiased questions, and uses psychometric properties such as difficulty and discrimination indices. Feedback mechanisms and technological integration, such as computer-based testing, enhance efficiency and provide detailed performance insights. For faculty members, creating excellent, faultless multiple-choice questions remains a challenge, especially for those without professional training. It has been observed that formal faculty training improves MCQ authoring abilities and MCQ quality.

Ethical statement

Ethical approval for the Research project number MEDA-2023-12-2356 was taken from the Bioethics Committee of the Northern Border University, Saudi Arabia. Informed consent was obtained from all the participants.

## Results

In this study, we analyzed the participants’ responses to pre- and post-workshop questionnaires. This revealed that the workshop was indeed helpful for the attendees. However, the participants unanimously felt that repeated or continuous FDPs are needed to bring significant improvement.

**Figure 1:**
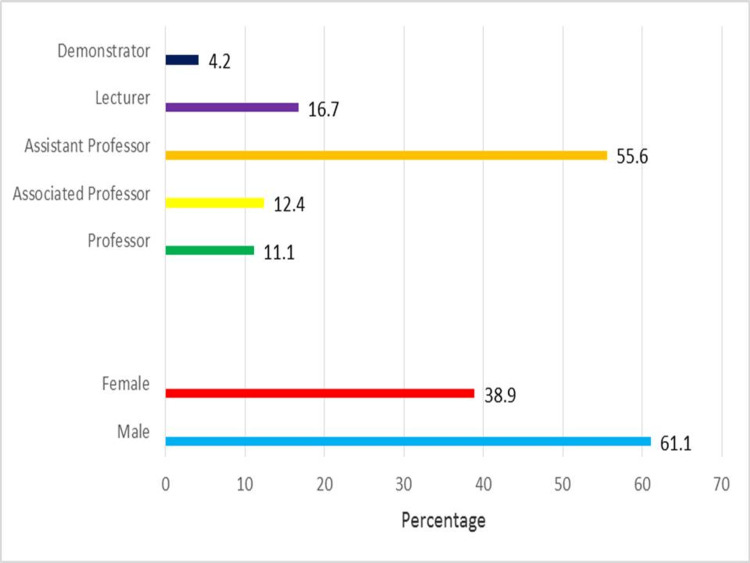
Participant characteristics

The workshop was attended by 56 medical faculty members. Participants included professors, associate professors, assistant professors, lecturers, and demonstrators from different disciplines of professional health colleges. The gender distribution and statistics of the participants’ designations are shown in Figure 1. There were 34 (61.1%) male participants in comparison to 22 (38.9%) female participants. When considering designation, the majority of participants were assistant professors 31 (55.6%).

For a simplified data analysis, the results were categorized as positive (strongly agree and agree), neutral, and negative (disagree and strongly disagree). Responses are presented as percentages.

Pre-workshop Questionnaire

The results of the pre-workshop questionnaire are detailed in Table 1. Most participants could not identify the most commonly occurring IWFs with high-quality MCQ writing. Although all of them were aware of the incidence of flaws, from the responses received, it can be understood that the participants were unable to identify the nature of these flaws. For example, the use of distractors and absolute negatives was considered tolerable by almost a quarter of the participants. Furthermore, 22 (38.9%) of participants felt that it was admissible to have more than one suitable option. Only 30 (53.7%) of the participants thought it was important to focus on technical interventions for faculty members for MCQ writing. Additionally, 38 (67.3%) perceived that their current skills were sufficient to write a well-constructed MCQ.

**Table 1:** Pre-workshop questionnaire results

	Questions/Statements	Agree	Disagree	Neutral
1	Occurrence of critical flaws during MCQ writing.	100	0	0
2	Need for focussed training for medical faculty in regard to MCQ writing.	53.7	26.9	19.4
3	Current MCQ writing skills are sufficient.	67.3	13.9	18.8
4	Negative questions are bad indicators.	50.3	5.6	44.1
5	‘Except’ marked questions should not be used.	57	11.1	31.9
6	Absolute and ambiguous terms should be avoided.	61.8	11.4	26.8
7	There should be no repetition of STEM words.	60.1	13.6	26.3
8	Answer options should not be heterogenous.	58.6	22.2	19.2
9	Unfavorable to have a single long option as the correct answer.	78.3	15.6	6.1
10	‘All of the above’ & ‘none of the above’ are bad indicators.	42.3	20.9	36.8
11	Options must be in chronological order.	55.6	33.9	10.5
12	It is okay to have more than a single suitable option.	59.2	11.9	28.9
13	Overlapping options can be present.	48.4	27.8	23.8
14	A vague or unclear STEM must not be used.	61.1	33.4	5.5
15	The presence of true or false type questions is good.	60.3	28.6	11.1
16	The usage of case scenarios irrelevant to the actual question is bad.	50.9	26.8	22.3

Post-workshop Questionnaire

**Figure 2:**
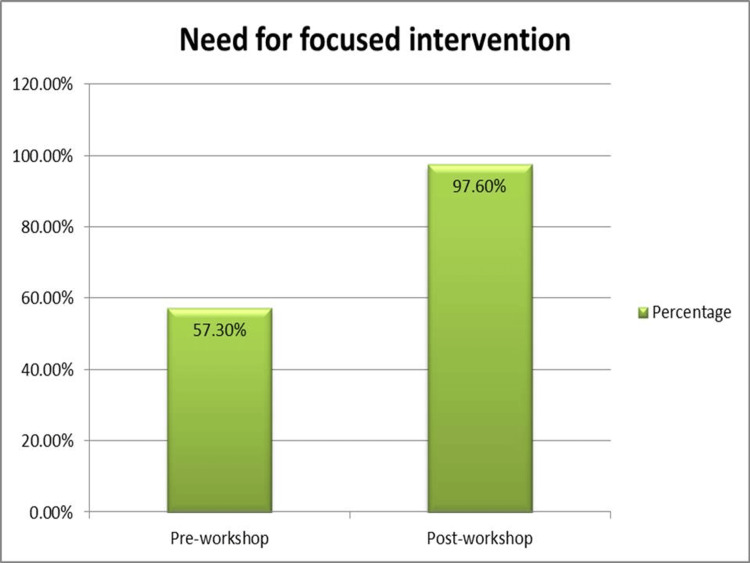
Attitude change toward focussed intervention

**Figure 3:**
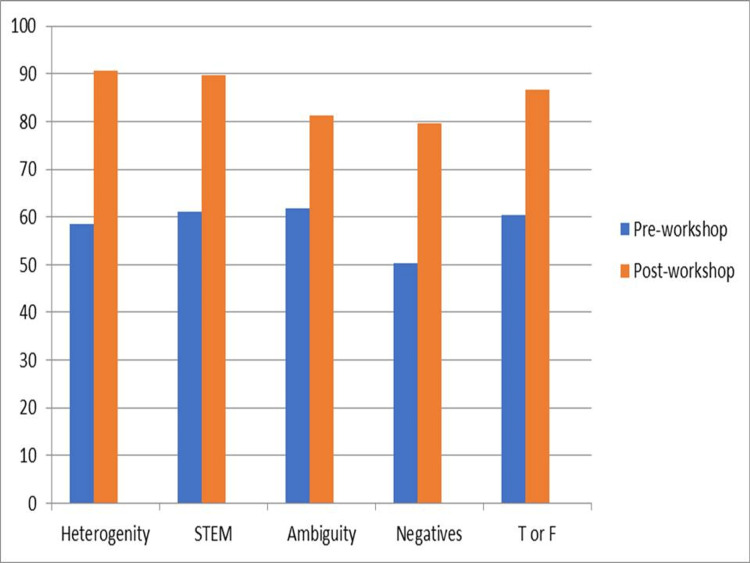
Post-workshop questionnaire data analysis

The analytics of the post-workshop questionnaire are presented in Table 2. This showed a major shift in the attitude of the participants towards focused interventions for MCQ writing and a significant improvement in how participants perceived IWFs. Most of the participants 47 (83.2%) agreed that the workshop would help them frame better MCQs with reduced cover test failure rates. The percentage of attendees who were able to successfully identify IWFs also increased significantly. Only 33 (58.6%) considered that their answers should not be heterogeneous before the session. However, it increased to 51 (90.6%) after the workshop. Most participants (67.3%) opined that the current workshop was beneficial with regard to the content delivered and the way it was organized. In addition, the percentage of participants who felt the need for focused training workshops to improve MCQ writing skills increased to 53 (94.6%) (Figures 2-3).

**Table 2:** Post-workshop test results

	Questions/Statements	Agree	Disagree	Neutral
1	Marked reduction in the flaws while MCQ framing following this training workshop.	78.6	18.3	3.1
2	Improvement in the quality of options used in MCQ writing.	88.9	9.2	1.9
3	Reduction in heterogeneity while designing MCQ with the help of the information obtained in this workshop.	90.6	4.3	5.1
4	Reduction in vover test failure rates after attending the workshop.	83.2	14.5	2.3
5	It is essential to frame a clear STEM and this workshop helped to improve the same.	89.8	8.7	18.9
6	Negative questions are bad indicators.	79.6	4.7	15.7
7	Ambiguous terms should be avoided.	81.3	15.1	3.6
8	Item analysis will reveal the improvement in the discrimination index and proportion of non-functioning distractors	90.8	7.6	1.6
9	A single, short-durational faculty training workshop will be helpful in improving the quality of MCQ writing	64.5	15.8	13.1
10	There is a need for focused training of medical faculty in the process of MCQ writing	94.6	4.1	1.3
11	Repeated or continuous FDP is needed to bring significant improvement.	85.1	12.2	2.7
12	It is possible to assess the problem-solving and reasoning of the students through MCQ.	88.2	6.9	4.9
13	Good-quality MCQ instigates critical thinking, proper interpretation, integration, synthesis and analysis of medical knowledge and facts.	81.9	2.8	0.9
14	Properly framed MCQs can differentiate high- and low-performing students.	79.9	12.5	7.5
15	High-level competency MCQs can change learning behavior among students.	93.7	1.1	1.8
16	High-quality MCQs can be used to test broader-curriculum content, are objective, reliable valid and enable ease of scoring.	91.6	5.6	2.8

The session successfully helped the participants understand the shortcomings in their MCQ writing and helped them understand the need for future interventions to improve their skills. The educational implications of this study were to encourage faculty members to make good-quality MCQs and to get an insight into faculty members who have knowledge and are willing to learn how to construct high-quality MCQs. Training workshops were also to help them refine their questions' STEM and item writing abilities and their capacity to identify and correct errors in making good-quality MCQs in their respective domains. Another implication of it was also to try to make a change in faculty members' attitudes towards their competency in MCQ construction following the workshop.

## Discussion

The MCQs' format is one of the most common tools of assessment used at almost all levels and across all specialties for both formal certification and competitive examinations. They are preferred because of their objectivity and ease of scoring a large number of students at a time. In addition to ensuring fairness, well-constructed MCQs can differentiate between high- and low-performing students. Thus, correct framing of the MCQ (also known as an item) is essential. Most faculty members are either not acquainted with standard MCQ guidelines or refrain from changing practices [12].

There is a school of thought that propagates the development of dedicated FDTPs, where the focus is on high-quality content, practice, feedback, and improvement. The involvement of expert medical educationists is pitched for the same. Longitudinal FDTPs are resource-intensive, apart from committing a longer duration of faculty time. However, short-duration FDTPs have not been adequately evaluated in terms of their functional output. Repeated short-course training has been shown to improve test preparation. An initial three-hour session followed by two-hour sessions over a period of three months was conducted as a study. This breakdown and repetition of high-quality sessions containing item analysis and feedback discussion resulted in improved item-writing skills in the faculty [13].

FDTWs should be perceived by faculty as an enriching experience that helps them build their academic skills. The present study utilized participant responses from the pre- and post-workshop questionnaires to determine if FDTWs organized for training faculty in writing MCQs were beneficial and if there was a need for such workshops in the future. The participants’ responses indicated that, although the single-day training session was satisfactory and useful, further training sessions would be helpful. This is in accordance with the findings of Sezari et al. [14].

In line with other similar studies, participants’ knowledge levels regarding MCQ framing showed significant improvement following the workshop, as evident from the results of the post-workshop questionnaire [7,14]. In short, a single, short training workshop on MCQ writing is both useful and informative for medical instructors. To understand the long-term impact of the workshop, the writing skills of the participants can be assessed at the end of the academic year [16].

Through the participants’ feedback, the need to conduct further workshops can be understood. Scot et al. [23] demonstrated how a brief intervention in the form of a lecture brought about short-term improvement in the quality of MCQ writing [23]. To ensure that this improvement is consistent and reliable, further faculty development workshops aimed at improving MCQ writing skills must have a proper structure and be organized at regular intervals [5]. The effect of continuous long-term FDTW was demonstrated in a study conducted by Abdulghani et al. [6]. An improvement in the performance of borderline students was observed after a longitudinal faculty development workshop aimed at improving the MCQ writing skills of the teaching faculty [6].

We believe that, through this study, we have shown that well-planned and intern training workshops can indeed be beneficial to the participants and can help them make a difference in the quality of their MCQ writing. The results of the post-workshop questionnaire indicate that the FDTW has given them ideas on how to reduce IWFs and, consequently, cover failure rates. In addition, shorter sessions have the advantage of being less labor-intensive and time-consuming. They can encourage staff participation and involvement owing to their concise nature and convenience. However, shorter sessions must be organized more frequently to reinforce the principles and guidelines to be followed [24]. Moore’s Framework, Bloom’s taxonomy, and Miller’s pyramid can be used to further support teachers’ ability to construct high-quality MCQs [25]. However, the quality of multiple-choice questions has been a source of concern because badly constructed questions can produce misleading evaluation results. Consequently, it is essential to ensure that the questions are properly constructed, in line with the learning objectives, and evaluate skills related to higher-order thinking [26]. A well-designed MCQ ought to evaluate a student's critical thinking and clinical reasoning skills, in addition to properly assessing the depth of their knowledge. MCQs should be rooted in clearly defined learning objectives pertinent to the curriculum and reflective of real-life medical scenarios to ensure good quality [27].

According to our research, developing MCQs of outstanding quality is a difficult task that requires ongoing learning and guidance. Although the one-day workshop outcomes analyzed in a few studies had little impact on the quality of MCQs making, further studies reported that one-day workshops were also beneficial for improving the quality of MCQ-based assessments [5,16]. Our results indicate that FDTWs as an intervention had a positive effect on participants' viewpoints, perceptions, and changes in attitude towards writing good MCQs, which was reflected in subsequent students' assessments of various courses taken by the students. One of the potential confounding variables is the duration and frequency of conducting workshops, which may affect study results. Therefore, we have added them as one of the study limitations, in addition to a small sample size of participants. Limitations of our study were mainly that the data from just a few short-duration workshops were included, not a large number of MCQs were examined, and faculty members of not all disciplines took part in the MCQ workshops. We are still continuing with faculty development training workshops on MCQ making. Further studies will focus on addressing these confounding variables in depth. Ongoing studies using a larger sample and more training sessions can give a better insight into research objectives.

Limitations

Data from only two workshops and a small number of MCQs were analyzed, with limited participation from faculty members. The study's limitations include its small sample size and short duration, suggesting that larger, more extensive studies are needed for better insights into the research objectives. This initial study introduces participants to MCQ design principles but does not assess long-term implementation, indicating a need for further investigation. The study shows that FDTWs can positively impact participants' perspectives and skills, but follow-up studies with larger samples are necessary for a more comprehensive understanding. Frequent, well-structured FDTWs are essential for continuous improvement, empowering faculty to create MCQs that assess students' clinical knowledge effectively. Despite its limitations, this study is a significant step towards enhancing the academic skills of medical educators.

## Conclusions

The results of this study help us understand that faculty development workshops and training workshops can help medical teaching faculty improve their skills while creating MCQs for student assessment. This is evident from the fact that, when the participating faculty members were introduced to Bloom's taxonomy, item analysis, and creation of proper STEM, their perception towards designing MCQs changed. They reported that attending the workshop helped them understand the process of producing quality questionnaires and will help them reduce the cover test failure rates in the future. However, continuous training workshops with longer durations or shorter durations are frequently conducted to improve the competence of the participants in writing high-quality MCQs. These workshops will be places where the participants will be able to work on the areas of improvement required to make a permanent impact. Training workshops can be evaluated using Kirkpatrick’s model to ensure that the objectives are fulfilled. A properly designed MCQ can help students avoid recall from memory, as well as problem-solving and reasoning. Hence, training teachers to frame MCQs correctly must be prioritized. We firmly believe that this will ensure that only high-quality MCQs are delivered to the student, ensuring the reliability and validity of the assessments.
